# Neuronal Injury and Regeneration-Linked Gene Expression Dynamics in the Hypothalamic–Pituitary–Adrenal Axis Following Experimental Traumatic Brain Injury

**DOI:** 10.3390/ijms27125172

**Published:** 2026-06-07

**Authors:** Halil Ulutabanca, Serhat Albayrak, Zeynep Yilmaz Şükranli, Begüm Er, Eray Abat, Serpil Taheri

**Affiliations:** 1 Department of Neurosurgery, Medical School, Erciyes University, Kayseri 38280, Turkey; 2 Betul Ziya Eren Genome and Stem Cell (GENKOK) Center, Kayseri 38280, Turkey; albyrkserhat41@gmail.com (S.A.); zeynepyilmaz_55@hotmail.com (Z.Y.Ş.); begumer@erciyes.edu.tr (B.E.); serpiltaheri@hotmail.com (S.T.); 3 Department of Medical Biology, Medical School, Erciyes University, Kayseri 38280, Turkey; 4 Department of Neurosurgery, Develi Dr. Ekrem Karakaya State Hospital, Develi, Kayseri 38400, Turkey; eray_abat@hotmail.com

**Keywords:** traumatic brain injury (TBI), hypothalamic–pituitary–adrenal (HPA) axis, gene expression, neuronal regeneration, real-time PCR, rat model

## Abstract

Traumatic brain injury (TBI) induces complex molecular and neuroendocrine alterations that extend beyond the site of injury. The hypothalamic–pituitary–adrenal (HPA) axis, a hierarchically organized neuroendocrine system composed of the hypothalamus, pituitary gland, and adrenal glands, plays a central role in coordinating stress and metabolic homeostasis. Despite its critical importance, the temporal transcriptional mechanisms underlying HPA axis dysregulation following TBI remain poorly understood, particularly in relation to coordinated neuronal injury and regeneration processes. This study aimed to investigate the time-dependent transcriptional dynamics of genes associated with neuronal injury and regeneration within the HPA axis following experimental TBI. Moderate-to-severe TBI was induced in Sprague–Dawley rats using a controlled cortical impact (CCI) model. Animals were allocated into sham, acute (24 h), and chronic (30 days) groups. Transcript profiles of 24 HPA axis- and neuroregeneration-related genes were analyzed in hypothalamic, pituitary, and adrenal tissues using quantitative real-time PCR, with normalization to a housekeeping gene and statistical evaluation of differential expression across time points. TBI induced distinct, tissue-specific, and time-dependent transcriptional alterations across the HPA axis. In the acute phase, stress-response genes showed divergent regulation between central and peripheral tissues, whereas the chronic phase was characterized by transcriptional reorganization involving neurotrophic, metabolic, and neuroendocrine pathways. Key regulators such as *Hif1a*, *Rad18*, *Avp*, *Gata3*, and *OxtR* exhibited significant and region-specific expression changes. These findings demonstrate that TBI triggers coordinated yet heterogeneous transcriptional responses within the HPA axis, linking central injury to systemic endocrine adaptation. This study provides novel insight into the molecular basis of neuroendocrine dysfunction and recovery after TBI and identifies candidate targets for future therapeutic strategies.

## 1. Introduction

Traumatic brain injury (TBI) is a non-degenerative brain insult caused by external mechanical forces, resulting in temporary or permanent neurological dysfunction and it remains a major global public health concern due to its substantial contribution to morbidity and mortality, particularly among young adults [1,2]. Head injuries account for a substantial proportion of trauma-related deaths, with many fatal cases occurring before patients reach medical care, emphasizing the continued need for improved preventive and therapeutic strategies [3].

The pathophysiology of TBI is broadly categorized into two phases: primary and secondary injury. The primary injury refers to the immediate mechanical damage sustained at the time of impact and currently lacks a targeted therapeutic approach. In contrast, secondary injury evolves through interconnected mechanisms, including ischemia, excitotoxicity, oxidative stress, inflammation, and mitochondrial dysfunction, observed in both experimental models and clinical settings [4,5]. These processes contribute to a cascade of detrimental events, such as cerebral edema, disruption of the blood–brain barrier (BBB), increased intracranial pressure, metabolic impairment, and the activation of apoptotic or necrotic pathways, ultimately leading to progressive neurological dysfunction [6,7,8]. Cerebral edema is one of the earliest manifestations of this network and may arise through both vasogenic mechanisms, driven by blood–brain barrier (BBB) permeability and extravascular fluid accumulation, and cytotoxic mechanisms, involving intracellular water retention, Na^+^/K^+^ pump dysfunction, oxidative stress, and mitochondrial impairment [9,10,11,12,13].

At the molecular level, hypoxia, oxidative stress, inflammatory activation, and DNA damage represent major organizing features of secondary injury. Hypoxia-inducible factor-1α (HIF-1α) coordinates transcriptional adaptation to reduced oxygen availability and has been implicated in neuronal apoptosis and central nervous system injury responses, although its role in promoting tissue damage versus recovery remains context-dependent [14,15,16]. In parallel, excessive production of reactive oxygen and nitrogen species promotes lipid peroxidation, protein oxidation, DNA damage, mitochondrial failure, and cell death [17,18,19,20]. DNA lesions, including single- and double-strand breaks, oxidative base modifications, and R-loop-associated damage, have been reported in experimental TBI and may disrupt transcription, cell-cycle regulation, inflammatory signaling, and neuronal survival if not adequately repaired [21,22,23,24,25,26,27,28,29].

Inflammation represents a key component of secondary injury following TBI, characterized by the activation of both cellular and humoral responses in reaction to disrupted tissue homeostasis. This response can exacerbate tissue damage beyond the primary insult through the release of mediators such as cytokines, chemokines, proteases, and reactive oxygen species (ROS), thereby amplifying injury propagation [30,31]. The arachidonic acid/eicosanoid pathway is particularly relevant in this context. Cyclooxygenase isoenzymes COX-1 and COX-2 regulate prostanoid production and influence vascular tone, leukocyte trafficking, microglial activation, and oxidative stress [32]. Experimental and post-mortem studies indicate that Ptgs2/Cox2 is dynamically induced after TBI, while Ptgs1/Cox1-expressing microglia/macrophages may persist in perilesional regions, suggesting phase-dependent and nonredundant roles for both isoforms in post-traumatic inflammation [33,34,35,36,37]. Counter-regulatory metabolic pathways may also shape this response; for example, Peroxisome proliferator-activated receptor alpha (PPAR-α) coordinates fatty-acid oxidation and anti-inflammatory transcriptional programs, and its pharmacological activation has been associated with reduced lesion burden and improved recovery in experimental TBI [38,39,40].

Although these local injury mechanisms are well recognized, TBI also disrupts systemic homeostatic circuits, particularly the hypothalamic–pituitary–adrenal (HPA) axis. The HPA axis coordinates the endocrine stress response through hypothalamic corticotropin-releasing hormone (CRH) and arginine vasopressin (AVP), pituitary proopiomelanocortin (Pomc)-derived adrenocorticotropic hormone, and adrenal glucocorticoid output. Structural injury, ischemia, inflammation, or secondary neurochemical disturbances involving hypothalamic and pituitary regions can impair this regulatory axis and contribute to cognitive, metabolic, immune, and endocrine abnormalities after TBI [41,42,43,44,45]. Clinical and experimental evidence further suggests that HPA-axis dysfunction may associate with systemic inflammatory signals, vasopressor dependence, and persistent post-traumatic endocrine abnormalities, supporting its relevance as an axis-level target of investigation rather than a peripheral consequence of brain injury [46,47,48,49].

In this study, we conceptualize post-traumatic HPA-axis dysfunction not as a uniform stress response, but as a distributed and temporally asynchronous transcriptional reprogramming across hypothalamic, pituitary, and adrenal compartments. This systems-level view is important because the acute phase of TBI may be dominated by hypoxia, edema, excitotoxicity, inflammatory amplification, and glucocorticoid stress signaling, whereas the chronic phase may reflect persistent or compensatory changes in DNA repair, trophic support, neuroimmune regulation, vascular remodeling, and endocrine feedback. Such a model predicts that transcriptional responses in one HPA-axis tissue may not be mirrored in another, and that acute molecular activation may evolve into chronic suppression, compensation, or maladaptive remodeling.

To test this framework, the selected transcriptional panel was organized around functional modules rather than isolated gene markers. The HPA-axis module included *Crh*, *Avp*, *Pomc*, and *corticosterone (Cort)* output to capture upstream hypothalamic drive, pituitary precursor biology, and adrenal glucocorticoid response. Cellular stress and metabolic adaptation were represented by *Hif1*α and Ppar-α, reflecting hypoxia sensing, energetic stress, lipid metabolism, and inflammatory resolution. DNA damage response and genomic stability were assessed through *Tert* and *Rad18*, which are relevant to telomere maintenance, oxidative resilience, PCNA ubiquitination, and lesion-bypass repair capacity [50,51,52,53,54]. Calcium-dependent signaling was represented by *Calm2*, given the role of calcium overload and calmodulin-dependent signaling in excitotoxicity, synaptic dysfunction, and post-traumatic signaling perturbation [55,56].

The panel also included genes linked to neural repair, plasticity, and circuit-level recovery. *SRY-Box Transcription Factor 2 (Sox2)* was selected as a regulator of neural stem/progenitor competence, while *Brain-derived neurotrophic factors (Bdnf)*, *Nerve growth factor (Ngf)*, *Fibroblast growth factor 2 (Fgf-2)*, and *Insulin-like growth factor-1 (Igf-1)* were included because neurotrophic and growth-factor signaling support neuronal survival, synaptogenesis, neurogenesis, gliogenesis, and post-injury remodeling [57,58,59,60,61,62]. *Oligodendrocyte transcription factor-2 (Olig-2)* was included as an oligodendroglial lineage regulator relevant to remyelination and white-matter restoration, which are increasingly recognized as important components of functional recovery after TBI [63]. *Apolipoprotein E (ApoE)* and *Angiotensin II receptor type 2 (Agtr2)* were incorporated as lipid-transport and vascular–neuroregulatory nodes, respectively, linking membrane repair, synaptic maintenance, vascular tone, neuroprotection, and neurogenesis to the broader post-traumatic response network [64,65,66,67,68,69].

Finally, neuroimmune and neuromodulatory components were included to capture the interface between inflammation, stress responsivity, and behavioral sequelae. *GATA binding protein 3 (Gata3)* was selected as a transcriptional regulator relevant to immune polarization within the integrated neuroimmune stress landscape [55,70]. *Oxytocin (Oxt)* and *Oxytocin receptor (Oxtr)* were included because oxytocinergic signaling modulates social behavior circuitry, stress adaptation, and neuroimmune processes, and has emerged as a candidate therapeutic axis for post-TBI social dysfunction [71,72,73]. Together, these modules provide a structured framework for evaluating how TBI affects endocrine regulation, inflammatory signaling, cellular stress responses, and regenerative plasticity across the HPA axis.

Therefore, this study aimed to characterize the acute and chronic transcriptional profiles of 23 genes related to HPA-axis regulation, cellular stress responses, neuroinflammation, neuromodulation, and neuronal regeneration in rat hypothalamus, pituitary, and adrenal tissues following experimental TBI. By comparing 24 h and 30-day post-injury phases, we sought to determine whether TBI produces tissue-specific and phase-dependent molecular signatures consistent with asynchronous HPA-axis reprogramming. This approach may help identify candidate biomarkers and mechanistic targets for TBI-induced neuroendocrine dysfunction.

## 2. Results

### 2.1. Gene Expression Alterations in the Hypothalamus Following TBI

**Figure 1:**
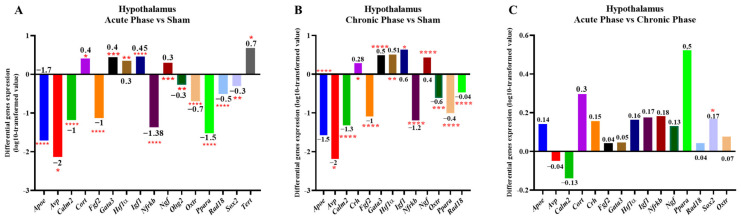
Gene expression levels (log10 transformed value) in hypothalamus. (**A**) Genes significantly altered between acute and sham groups (adj. *p* < 0.05). (**B**) Genes significantly altered between chronic and sham groups (adj. *p* < 0.05). (**C**) Genes significantly altered between acute and chronic groups (adj. *p* < 0.05). The sham control group corresponds to a baseline value of 0 after transformation. Statistical significance is denoted by asterisks (* adj. *p* < 0.05, ** adj. *p* < 0.01, *** adj. *p* < 0.001, **** adj. *p* < 0.0001).

Following trauma, the acute and chronic phases in the hypothalamus were compared with the sham control group, and additionally, the acute and chronic periods were compared with each other. During the acute phase, *Rad18* (*p* = 0.000005), *Nfrkb* (*p* = 0.000006), *Ppara* (*p* = 0.000028), *Apoe* (*p* = 0.000031), *Fgf2* (*p* = 0.000036), *Calm2* (*p* = 0.000245), *Oxtr* (*p* = 0.000520), *Olig2* (*p* = 0.002654), *Sox2* (*p* = 0.002793), and *Avp* (*p* = 0.026808) were significantly downregulated compared with the sham control group. In contrast, *Igf1* (*p* = 0.000141), *Gata3* (*p* = 0.001644), *Ngf* (*p* = 0.001874), *Hif1α* (*p* = 0.004877), *Cort* (*p* = 0.013198), and *Tert* (*p* = 0.016790) were significantly upregulated in the acute period relative to sham controls. Although *Bdnf*, *Pomc*, *Crh*, *Oxt*, *Ptgs2*, *Ptgs1*, and *Agtr2* also showed expression changes, these differences were not statistically significant (Table 1, Figure 1A). Overall, these findings indicate a marked but mixed transcriptional response in the hypothalamus during the acute phase following TBI, characterized by the predominant downregulation of genes associated with metabolic, glial, and neuroendocrine signaling, accompanied by selective upregulation of stress- and trophic-response genes.

During the chronic period, compared with the sham control group, *Rad18* (*p* = 0.000015), *Nfrkb* (*p* = 0.000016), *Apoe* (*p* = 0.000063), *Fgf2* (*p* = 0.000073), *Ppara* (*p* = 0.000320), *Calm2* (*p* = 0.000325), *Oxtr* (*p* = 0.001438), and *Avp* (*p* = 0.032760) were significantly downregulated in the hypothalamus. Among these, the most marked negative changes were observed for *Avp*, *Fgf2*, and *Oxtr*, indicating substantial suppression of neuroendocrine and trophic signaling during the chronic phase. In contrast, *Gata3* (*p* = 0.000740), *Ngf* (*p* = 0.000832), *Hif1α* (*p* = 0.007128), *Igf1* (*p* = 0.015516), and *Crh* (*p* = 0.036870) were significantly upregulated relative to the sham control group. The strongest increases were observed for *Gata3* and *Hif1a*, suggesting sustained transcriptional activation of stress-, immune-, and adaptive-response pathways in the chronic phase (Table 2, Figure 1B).

Comparing gene expression changes between the acute and chronic phases in the hypothalamus, only *Sox2* showed a statistically significant increase in the chronic phase (*p* = 0.034809). Although *Ngf*, *Hif1α*, *Gata3*, and *Igf1* also displayed higher expression in the chronic period compared with the acute period, these differences did not reach statistical significance. Overall, these findings indicate that while the chronic hypothalamic response retains the broad transcriptional pattern observed in the acute phase, it is distinguished by selective enhancement of *Sox2* expression rather than widespread additional transcriptional remodeling (Figure 1C).

### 2.2. Gene Expression Alterations in the Pituitary Following TBI

**Figure 2:**
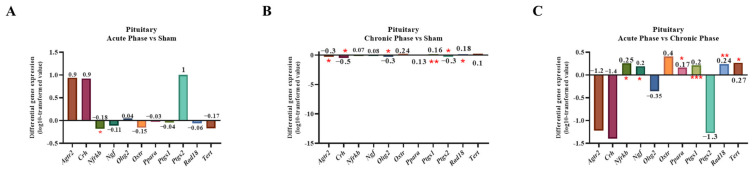
Gene expression levels (log10 transformed value) in the pituitary. Gene expression levels (log10 transformed value) in the pituitary. (**A**) Genes significantly altered between acute and sham groups (adj. *p* < 0.05). (**B**) Genes significantly altered between chronic and sham groups (adj. *p* < 0.05). (**C**) Genes significantly altered between acute and chronic groups (adj. *p* < 0.05). The sham control group corresponds to a baseline value of 0 after transformation. Statistical significance is denoted by asterisks (* adj. *p* < 0.05, ** adj. *p* < 0.01, *** adj. *p* < 0.001).

Following TBI, the acute and chronic phases in the pituitary were compared with the sham control group, and additionally, the acute and chronic periods were compared with each other. In the acute phase, *Nfrkb* was significantly downregulated compared with the sham control group (*p* = 0.0159), whereas the changes observed in *Ptgs1*, *Rad18*, *Tert*, *Ngf*, *OxtR*, *Ppara*, and the other analyzed genes did not reach statistical significance (Figure 2A). These findings indicate that the pituitary exhibits only a limited early transcriptional response following TBI.

In contrast, comparison of the chronic phase with the sham control group revealed a broader pattern of transcriptional alteration. *Ptgs1* (*p* = 0.0052) and *Rad18* (*p* = 0.0265) were significantly upregulated, whereas *Crh* (*p* = 0.0111), *Ptgs2* (*p* = 0.0240), *Agtr2* (*p* = 0.0432), and *Olig2* (*p* = 0.0466) were significantly downregulated (Figure 2B). These findings suggest that, unlike the relatively muted acute response, the chronic phase is characterized by a more distinct transcriptional remodeling in the pituitary.

Consistent with this pattern, direct comparison of the chronic and acute phases demonstrated significant upregulation of *Ptgs1* (*p* = 0.0018), *Rad18* (*p* = 0.0082), *Nfrkb* (*p* = 0.0126), *Tert* (*p* = 0.0199), *Ppara* (*p* = 0.0201), *Oxtr* (*p* = 0.0202), and *Ngf* (*p* = 0.0359) in the chronic period relative to the acute phase (Table 3, Figure 2C). Among these, *Rad18* showed one of the most prominent increases, supporting the presence of an amplified late response involving DNA damage-associated and adaptive transcriptional pathways. Overall, these results indicate that pituitary gene expression changes become more apparent during the chronic phase after TBI, reflecting delayed and selective molecular remodeling rather than marked early disruption.

### 2.3. Gene Expression Alterations in the Adrenal Glands Following TBI

**Figure 3:**
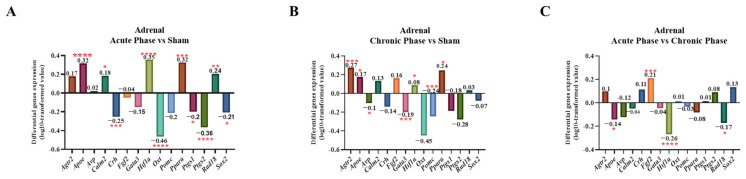
Gene expression levels (log10 transformed value) in the adrenal. Gene expression levels (log10 transformed value) in the adrenal. (**A**) Genes significantly altered between acute and sham groups (adj. *p* < 0.05). (**B**) Genes significantly altered between chronic and sham groups (adj. *p* < 0.05). (**C**) Genes significantly altered between acute and chronic groups (adj. *p* < 0.05). The sham control group corresponds to a baseline value of 0 after transformation. Statistical significance is denoted by asterisks (* adj. *p* < 0.05, ** adj. *p* < 0.01, *** adj. *p* < 0.001, **** adj. *p* < 0.0001).

Following TBI, the acute and chronic phases in the adrenal glands were compared with the sham control group, and additionally, the acute and chronic periods were compared with each other. In the acute phase, *Hif1α* (*p* = 0.000014), *Apoe* (*p* = 0.000070), *Ppara* (*p* = 0.000415), *Rad18* (*p* = 0.002745), and *Calm2* (*p* = 0.019750) were significantly upregulated compared with the sham control group, whereas *Ptgs2* (*p* = 0.000232), *Oxt* (*p* = 0.000274), *Crh* (*p* = 0.001810), *Sox2* (*p* = 0.015503), and *Ptgs1* (*p* = 0.019239) were significantly downregulated (Table 4, Figure 3A). This pattern reflects a robust early transcriptional response involving both activation and suppression of key regulatory pathways.

In the chronic phase, comparison with the sham control group showed significant upregulation of *Agtr2* (*p* = 0.000345), *Apoe* (*p* = 0.010422), *Hif1α* (*p* = 0.011217), and *Ppara* (*p* = 0.017408), whereas *Gata3* (*p* = 0.001449), *Pomc* (*p* = 0.003199), and *Avp* (*p* = 0.031612) were significantly downregulated (Table 5, Figure 3B). Unlike the acute phase, genes associated with prostaglandin signaling were no longer significantly altered, while endocrine- and regulatory-related genes became more prominent. These findings suggest a shift in the adrenal transcriptional response during the chronic phase.

Comparison of gene expression changes between the acute and chronic phases demonstrated significant downregulation of *Hif1α* (*p* = 0.000180), *Rad18* (*p* = 0.004365), and *Apoe* (*p* = 0.018273) in the chronic phase, whereas *Fgf2* (*p* = 0.001647) was significantly upregulated (Table 6, Figure 3C). Together, these findings indicate a transition from an early response characterized by hypoxia-associated, calcium-dependent, and DNA damage-related signaling toward a later phase marked by reduced acute stress signaling and increased trophic or remodeling-related activity.

In the acute phase, the upregulation of *Hif1α*, *Ppara*, *Rad18*, *Apoe*, and *Calm2* indicates activation of hypoxia-responsive, metabolic, DNA damage-associated, and calcium-dependent signaling pathways, while the downregulation of *Ptgs1*, *Ptgs2*, *Oxt*, *Crh***,** and *Sox2* suggests suppression of prostaglandin-related, neuroendocrine, and transcriptional regulatory mechanisms under early post-traumatic conditions. In the chronic phase, persistent upregulation of *Apoe*, *Hif1α*, and *Ppara*, together with increased *Agtr2* and decreased *Gata3*, *Pomc*, and *Avp*, reflects a reorganization of adrenal gene expression toward longer-term metabolic and endocrine adaptation. Overall, these findings support the view that the adrenal gland undergoes substantial temporal transcriptional remodeling following TBI, with distinct molecular signatures in the acute and chronic periods.

## 3. Discussion

### 3.1. General Overview of TBI Pathophysiology

Traumatic brain injury (TBI) is characterized by primary mechanical damage followed by secondary processes, which proceed through a complex molecular network involving hypoxia, metabolic stress, inflammation, and DNA damage responses. These secondary cascades substantially influence long-term neurological and systemic outcomes. Given the high morbidity and mortality associated with TBI, identifying the molecular mechanisms underlying these processes remains essential for developing effective neuroprotective and therapeutic strategies.

Our findings indicate that changes in genes such as *Hif1a*, *Ppara*, and *Rad18* reflect the activation of mechanisms related to hypoxia response, metabolic reprogramming, and maintenance of genomic stability following TBI. In addition, the suppression of prostaglandin-mediated inflammatory signaling (*Ptgs1*, *Ptgs2*) suggests a reorganization of the classical inflammatory response in the early phase. This overall framework supports the concept that TBI is not merely a localized injury but a systemic and dynamic process of molecular remodeling.

### 3.2. HPA Axis Dysfunction Following TBI

Among the secondary consequences of TBI, alterations in the hypothalamic–pituitary–adrenal (HPA) axis play a pivotal role in the regulation of stress, metabolism, and immune responses. Disruption of this axis can result in hypopituitarism, adrenal insufficiency, and dysregulated glucocorticoid signaling, all of which contribute to impaired neuroendocrine homeostasis and poor recovery. Experimental and clinical studies have suggested that these dysfunctions may persist chronically, leading to sustained hormonal and behavioral disturbances even after apparent neurological recovery. When changes observed in the hypothalamus, pituitary, and adrenal glands are considered together, a marked dysregulation of the HPA axis emerges following TBI. In the hypothalamus, the significant reduction in *Avp* expression indicates suppressed neuroendocrine signaling in the chronic phase and a fundamental disruption of axis function. In the adrenal glands, genes associated with stress response are activated in the acute phase, whereas in the chronic phase, this response shifts toward metabolic and endocrine adaptation. In the pituitary, the initially limited response becomes more pronounced in the chronic phase, indicating asynchronous temporal involvement of axis components. These findings suggest that HPA axis dysfunction following TBI is not static but a dynamic, phase-dependent process.

### 3.3. Temporal and Tissue-Specific Transcriptional Responses

In the present study, we investigated temporal and tissue-specific gene expression dynamics of 24 genes associated with the HPA axis regulation, cellular stress responses, and neuronal regeneration in a rat model of TBI. Our data revealed that TBI induces distinct, region- and phase-dependent transcriptional responses in the hypothalamus, pituitary, and adrenal glands. Specifically, the acute phase (24 h) was characterized by early activation of oxidative and stress-response genes, whereas the chronic phase (30 d) demonstrated long-term transcriptional reorganization suggestive of compensatory or maladaptive remodeling within the HPA axis.

In the hypothalamus, the overall acute-phase transcriptional pattern is largely preserved in the chronic phase; however, rather than a broad reprogramming, selective changes predominate. In contrast, the pituitary exhibits a delayed but pronounced transcriptional activation, suggesting a late adaptive response. In the adrenal glands, the acute phase is characterized by a strong and bidirectional response (activation and suppression), whereas the chronic phase shifts toward tissue remodeling and metabolic adaptation. These tissue-specific and temporal differences support the concept that TBI induces a distributed and asynchronous molecular remodeling process rather than a uniform response.

**Figure 4:**
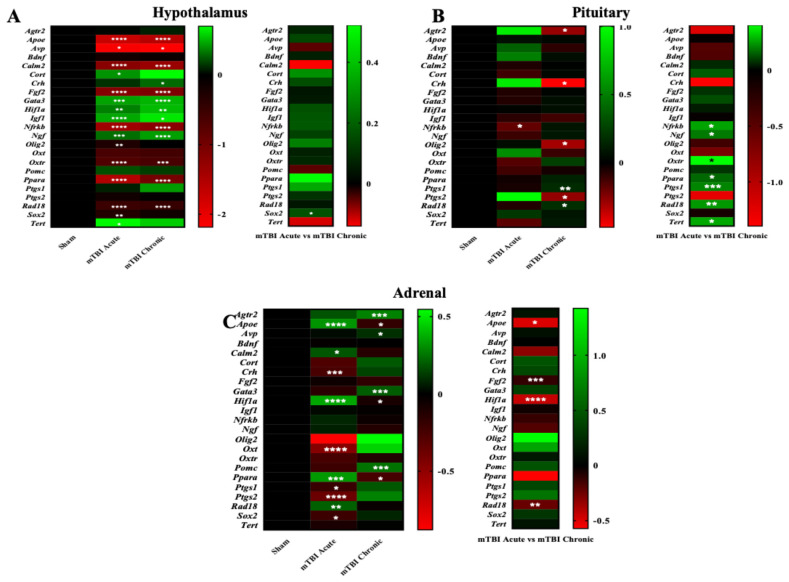
Heatmap representation of gene expression profiles in the hypothalamus, pituitary gland, and adrenal glands during the acute and chronic phases following trauma, based on values after normalization with log10 transformation applied. Green and red color gradients indicate upregulation and downregulation, respectively, with color intensity reflecting the magnitude of expression changes. Comparisons include sham versus acute phase, sham versus chronic phase, and acute versus chronic phase, highlighting temporal and tissue-specific transcriptional responses to trauma. The sham control group corresponds to a baseline value of 0 after transformation. Statistical significance is denoted by asterisks (* adj. *p* < 0.05, ** adj. *p* < 0.01, *** adj. *p* < 0.001, **** adj. *p* < 0.0001).

In the acute phase, the hypothalamus displayed a marked but mixed transcriptional response, with significant downregulation of *Rad18*, *Nfrkb*, *Ppara*, *Apoe*, *Fgf2*, *Calm2*, *Oxtr*, *Olig2*, *Sox2*, and *Avp*, alongside significant upregulation of *Igf1*, *Gata3*, *Ngf*, *Hif1α*, *Cort*, and *Tert*. In the chronic phase, this pattern persisted in part, as *Rad18*, *Nfrkb*, *Apoe*, *Fgf2*, *Ppara*, *Calm2*, *Oxtr*, and *Avp* remained significantly downregulated, whereas *Gata3*, *Ngf*, *Hif1α*, *Igf1*, and *Crh* were significantly upregulated. These findings support sustained hypothalamic dysregulation after TBI, particularly in neuroendocrine, trophic, and metabolic signaling pathways. In contrast, the pituitary showed only limited acute responsiveness, with significant downregulation of *Nfrkb* alone, whereas the chronic phase revealed clearer remodeling, including upregulation of *Ptgs1* and *Rad18* and downregulation of *Crh*, *Ptgs2*, *Agtr2*, and *Olig2*. Moreover, direct comparison of chronic and acute pituitary samples demonstrated significant increases in *Ptgs1*, *Rad18*, *Nfrkb*, *Tert*, *Ppara*, *Oxtr*, and *Ngf*, indicating a delayed pituitary response that becomes more evident over time. In the adrenal gland, the acute phase was characterized by significant upregulation of *Hif1α*, *Apoe*, *Ppara*, *Rad18*, and *Calm2* together with downregulation of *Ptgs2*, *Oxt*, *Crh*, *Sox2*, and *Ptgs1*, whereas the chronic phase showed upregulation of *Agtr2*, *Apoe*, *Hif1α*, and *Ppara* and downregulation of *Gata3*, *Pomc*, *and Avp.* The dramatic reduction in *Avp* expression is particularly consistent with persistent neuroendocrine dysfunction within the HPA axis long after the initial trauma. Comparison of chronic and acute adrenal samples further revealed decreased *Hif1α*, *Rad18*, and *Apoe* expression but increased *Fgf2*, consistent with temporal remodeling of adrenal stress and trophic signaling after injury. In the chronic phase, *Rad18* upregulation may reflect a sustained activation of DNA repair mechanisms in response to accumulated genomic stress. However, the concurrent alterations observed across hypothalamic and adrenal components suggest disruption of HPA axis feedback regulation. These findings point toward a progressive impairment of neuroendocrine balance rather than complete functional recovery (Figure 4).

Taken together, these results suggest that TBI induces not a uniform HPA-axis response, but a time-dependent and tissue-specific transcriptional reorganization. The hypothalamus appears to be the most transcriptionally responsive structure at both time points, with persistent suppression of several neuroendocrine and support-related genes despite selective activation of stress- and repair-associated pathways. The pituitary, by contrast, shows a comparatively delayed molecular response, becoming more transcriptionally active in the chronic period. The adrenal glands exhibit a clear temporal shift from acute hypoxia-, calcium-, and DNA damage-associated signaling toward a chronic profile more consistent with endocrine adaptation and tissue remodeling. Overall, this pattern is compatible with prolonged neuroendocrine disequilibrium after TBI rather than simple linear recovery.

These findings suggest that TBI induces not only localized brain tissue injury but also delayed and tissue-specific transcriptional responses within peripheral components of the HPA axis. The differential expression profiles of *Hif1α*, *Rad18*, *Avp*, *Gata3*, and *Oxtr* highlight their potential roles as molecular markers of both secondary damage and compensatory regeneration following TBI. Given the scarcity of studies investigating gene-level alterations in the HPA axis post-TBI, our findings contribute valuable insight into the systemic effects of TBI and may guide the identification of novel therapeutic targets (Figure 4).

Our findings support the view that TBI induces a time-dependent and tissue-specific reorganization of HPA-axis transcription, rather than a uniform endocrine response across all tissues. The predominance of hypothalamic changes in the acute phase is consistent with experimental evidence showing that TBI rapidly perturbs hypothalamic stress circuitry and activates the HPA axis early after injury [121], whereas pituitary dysfunction may be less apparent initially and can become clinically or molecularly more evident over time after trauma [122,123,124].

In this context, the persistent hypothalamic downregulation of *Avp*, *Oxtr*, *Fgf2*, *Ppara*, *Apoe*, *Calm2*, and other regulatory genes, together with the sustained upregulation of *Hif1α*, *Ngf*, *Gata3*, *Igf1*, and *Crh*, suggests that chronic post-traumatic adaptation occurs on a background of incomplete neuroendocrine recovery rather than restored homeostasis(Figure 4). This interpretation is strengthened by prior clinical evidence that posterior pituitary dysfunction and vasopressin-related abnormalities can persist after TBI, supporting the idea that reduced hypothalamic *Avp* expression may reflect enduring HPA-axis disequilibrium rather than a purely transient stress response [122].

By contrast, the pituitary in our study showed a relatively limited acute response but a clearer chronic-phase shift, with significant chronic-versus-acute increases in *Rad18*, *Nfrkb*, *Tert*, *Ppara*, *Oxtr*, and *Ngf*. This delayed profile is compatible with longitudinal human studies demonstrating that post-traumatic pituitary abnormalities may evolve dynamically, with some deficits resolving and others emerging or worsening during follow-up, rather than remaining static from the acute period onward [123,124,125].

The adrenal gland displayed yet another trajectory, characterized by acute induction of *Hif1α*, *Apoe*, *Ppara*, *Rad18*, and *Calm2*, followed by a chronic pattern marked by continued *Hif1α/Ppara/Apoe* elevation, *Agtr2* induction, and relative reduction in *Hif1α* and *Rad18* versus the acute phase(Figure 4).This temporal pattern is biologically plausible because *HIF-1α* has been shown to directly regulate adrenal steroidogenesis [118], while *ApoE* contributes to adrenal cholesterol storage and trafficking, processes that are central to steroid hormone synthesis [117,120].

More broadly, the direction of several injury-responsive genes in our dataset is in line with prior trauma literature showing that PPARα is induced in injured human brain tissue [126], *NGF* is upregulated after cortical trauma [127], FGF-2 participates in post-traumatic neurogenic and degenerative responses [77], and IGF-1/IGF-1R signaling undergoes temporal and regional alteration after brain injury [128]. Taken together, these observations argue that post-traumatic HPA-axis dysfunction should be viewed as a distributed and evolving molecular process, in which hypothalamic dysregulation is prominent, pituitary remodeling is delayed, and adrenal adaptation remains dynamically coupled to both injury burden and compensatory endocrine signaling.

### 3.4. Mechanistic Considerations of AVP and GATA3 Alterations

One important limitation of the present study is that the observed transcriptional changes cannot be directly attributed to a single underlying mechanism. However, several biologically plausible explanations may account for the marked alterations detected, particularly for *Avp* and *Gata3*.

The profound downregulation of *Avp* in the chronic phase may reflect multiple, non-mutually exclusive mechanisms. First, it may indicate selective vulnerability and potential loss or dysfunction of vasopressin-producing neurons within the hypothalamic paraventricular and supraoptic nuclei, which are known to be sensitive to ischemia, oxidative stress, and excitotoxic injury following TBI. Second, persistent suppression of *Avp* transcription may arise from epigenetic modifications, such as DNA methylation or histone remodeling, which have been increasingly implicated in long-term post-traumatic gene regulation. Third, altered HPA axis feedback dynamics, including prolonged glucocorticoid exposure, may suppress hypothalamic *Avp* expression via enhanced negative feedback signaling. These mechanisms are not mutually exclusive and may act in concert to produce the sustained neuroendocrine dysregulation observed in the chronic phase.

The marked and persistent downregulation of the *Avp* gene in the hypothalamus emerges as a key molecular component of HPA axis dysfunction following TBI. Reduced AVP signaling may contribute to impaired sustainability of the stress response and weakened neuroendocrine regulation. In contrast, the increase in *Gata3* expression, particularly in the chronic phase, suggests a shift in the immune response toward a Th2-associated profile and the establishment of a more regulatory and reparative microenvironment.

The opposing regulation of these two genes indicates that both neuroendocrine suppression and immune reprogramming occur simultaneously during the post-TBI course. Therefore, alterations in the *AVP* and *GATA3* axis can be considered important molecular markers representing the transition from acute injury response to the chronic adaptive phase.

Similarly, the striking upregulation of *Gata3* in the hypothalamus suggests a complex and potentially multifaceted biological response rather than a simple shift toward a Th2-like immune profile. While *Gata3* is classically associated with immune polarization, emerging evidence indicates that it also plays roles in neurodevelopmental transcriptional programs, cellular differentiation, and stress-responsive gene regulation. Its dramatic induction following TBI may therefore reflect the following: (i) reactivation of developmental transcriptional pathways as part of an attempted regenerative response; (ii) neuroimmune crosstalk, particularly involving microglia and infiltrating immune cells; or (iii) a broader transcriptional reprogramming state triggered by injury-induced signaling cascades. The magnitude of the observed change suggests that *Gata3* may function as a central regulatory node within the post-traumatic transcriptional network rather than as a passive marker of immune skewing.

### 3.5. Study Limitations and Future Directions

Taken together, these considerations highlight that the transcriptional alterations observed in this study likely arise from an interplay of cellular loss, epigenetic regulation, neuroendocrine feedback disruption, and injury-induced transcriptional reprogramming, underscoring the need for future studies integrating cell-type-specific and epigenomic analyses to delineate these mechanisms more precisely.

## 4. Materials and Methods

### 4.1. Ethical Approval

This study was conducted at the Betül-Ziya Eren Genome and Stem Cell Center (GENKOK), Erciyes University, following the approval of the Erciyes University Local Ethics Committee for Animal Experiments (Decision No.: 16/103, dated 27 July 2016). All procedures complied with international ethical standards for the care and use of laboratory animals.

### 4.2. Animals and Experimental Design

Male Sprague–Dawley rats (approximately 3 months old; 250–350 g) were used in the experiments. The animals were housed under controlled environmental conditions (22 ± 1 °C, 12 h light/dark cycle) with free access to standard pellet chow and water. Before the experiments, all animals underwent a one-week acclimatization period. A total of 42 rats were randomly divided into three experimental groups using a simple randomization method: (1) Sham control group: subjected to all surgical procedures except trauma induction and sacrificed; accordingly, (2) Acute TBI group: sacrificed 24 h post-injury; and (3) Chronic TBI group: sacrificed 30 days post-injury.

### 4.3. Induction of Traumatic Brain Injury

Traumatic brain injury (TBI) was induced using a computer-controlled Controlled Cortical Impact (CCI) device developed by Dr. Mehmet Bilgen. This system allows precise adjustment of impact parameters such as speed, depth, and duration, and was optimized to model severe human TBI characterized by focal cortical damage, hippocampal involvement, cerebral edema, and increased intracranial pressure.

Anesthesia was induced with 4% isoflurane and maintained with 2% isoflurane. Animals were positioned prone and stabilized in a stereotactic frame. Under aseptic conditions, a 10 mm midline scalp incision was made, and the periosteum was carefully dissected. A 3 mm craniotomy was created over the left parasagittal cortex, located 2 mm lateral to the sagittal suture and 2 mm posterior to bregma. The CCI was delivered using a 2.5 mm impactor tip with a depth of 1.5 mm, a velocity of 1.5 m/s, and a dwell time of 85 milliseconds. After the procedure, the scalp incision was closed with 3/0 silk sutures. Sham-operated animals underwent the same surgical procedure without impact.

### 4.4. Tissue Collection and RNA Extraction

Following TBI induction, rats in the acute group were sacrificed 24 h post-injury, and those in the chronic group were sacrificed 30 days post-injury. Sacrifice was performed by decapitation under ketamine anesthesia. The hypothalamus, pituitary gland, and adrenal glands were carefully dissected and immediately stored at −80 °C until molecular analysis. Total RNA was extracted using the TRIzol reagent (Bio-Rad Laboratories, Cat No: 7326890 Hercules, CA, USA), and complementary DNA (cDNA) was synthesized using the High-Fidelity cDNA Synthesis Kit (Roche Diagnostics GmbH, Cat No: 05091284001, Mannheim, Germany according to the manufacturer’s instructions.

### 4.5. Quantitative Real-Time PCR (qPCR)

Gene expression analysis was performed using Real-Time PCR on a Light Cycler 480 II system (Roche Diagnostics GmbH, Mannheim, Germany). A total of 24 genes associated with oxidative stress, DNA repair, neuroendocrine regulation, and neuronal regeneration were analyzed using the Probe Master Mix (Roche, Cat No:04707494001, Mannheim, Germany) in accordance with the manufacturer’s instructions, employing gene-specific primers and probes (Roche Diagnostics GmbH, Mannheim, Germany): *ApoE*, *Hif1α*, *Tert*, *Rad18*, *Sox2*, *Crh*, *Pomc*, *Cort*, *Oxt*, *Oxtr*, *Igf1*, *Fgf2*, *Avp*, *Agtr2*, *Gata3*, *Calm2*, *Bdnf*, *Ptsg1*, *Ptsg2*, *Olig2*, *Ngf*, and *Ppara*. The *β-actin* (*Actb*) gene served as the internal control (The specific catalog numbers of the primers are provided in the Supplementary Materials). All samples were run in duplicate. Relative gene expression was calculated using the 2^−ΔΔCT^ method after normalization to the housekeeping gene [1].

### 4.6. Statistical Analysis

All statistical analyses were conducted using R software version 3.2.0 (www.r-project.org). Data normality was assessed using the Shapiro–Wilk test and Q–Q plots, while Levene’s test evaluated homogeneity of variances. Data were collected using Real-Time PCR on a Light Cycler 480 II system (Roche), the linear derivative baseline correction method, and the auto global Cq threshold method. Median Limit of detection (LOD) Cq values were calculated across all arrays to impute missing values [129]. Data normalization was performed using the 2^−∆∆Ct^ method [130]. Following normalization, gene expression changes in the acute and chronic TBI groups were calculated relative to the sham control group as fold changes. Subsequently, these values were subjected to log10 transformation prior to downstream analyses [131]. Log10 transformation was applied to the raw expression values to address heteroscedasticity, which is commonly observed in high-throughput gene expression data where variance tends to increase with the mean [131]. By compressing the dynamic range of the data, this transformation approximates a more normal distribution, thereby satisfying the assumptions underlying many parametric statistical tests. In addition, log-scaled values allow fold-changes to be interpreted symmetrically—for instance, a tenfold increase and a tenfold decrease are represented as equal and opposite shifts—which improves both the interpretability and the comparability of results across genes. For comparisons between two groups, Student’s *t*-test was applied. All *p*-values were adjusted using the Benjamini–Hochberg false discovery rate correction to account for multiple testing, implemented in R Studio (version 4.5.3.; RStudio, Boston, MA, USA) and GraphPad Prism (version 8.0; GraphPad Software, San Diego, CA, USA).

The obtained data were statistically analyzed by calculating: (i) the changes in the acute TBI group compared to the sham control group, (ii) the changes in the chronic TBI group compared to the sham control group, and (iii) the differences between the chronic TBI group and the acute TBI group. *p*- and adjusted *p*-values < 0.05 were considered statistically significant.

## 5. Conclusions

This study comprehensively characterizes the temporal transcriptional landscape of genes associated with the hypothalamic–pituitary–adrenal (HPA) axis and neuronal regeneration in a rat model of traumatic brain injury (TBI). Our findings demonstrate that TBI triggers distinct, tissue-specific, and time-dependent transcriptional responses across the hypothalamus, pituitary, and adrenal glands.

Notably, the differential expression of key regulators such as *Hif-1α*, *Rad18*, *Avp*, *Gata3*, and *Oxtr* underscores their potential as molecular indicators of both neuroendocrine dysfunction and adaptive/regenerative processes following TBI. The observed transcriptional shifts highlight how secondary systemic responses extend beyond the central lesion, involving peripheral endocrine structures that contribute to the regulation of stress and metabolic homeostasis.

Together, these results provide novel insight into the dynamic and coordinated regulation of the HPA axis after brain injury, emphasizing the interplay between central and peripheral systems in post-traumatic adaptation. By identifying candidate genes implicated in both injury propagation and recovery, this study establishes a foundation for the development of targeted therapeutic strategies aimed at restoring neuroendocrine balance and improving long-term outcomes after TBI.

## Figures and Tables

**Table 1:** Log10 transformation value of gene expression in the hypothalamus between the acute TBI and sham groups. The sham control group corresponds to a baseline value of 0 after transformation.

Gene	Log10 Transformed Value	Adjusted *p*-Value (Adj. *p*. Val)	Regulation	Functional Relevance (Selected References)
***Rad18***	−0.5093	0.000005	↓ Downregulated	DNA repair E3 ligase; reduced expression reflects impaired post-replicative DNA repair after oxidative stress [54].
***Nfrkb***	−1.37264	0.000006	↓ Downregulated	Supports INO80-dependent chromatin remodeling and stress-responsive transcription; decreased expression suggests reduced transcriptional adaptability and DNA repair capacity [74]
***Ppara***	−1.52819	0.000028	↓ Downregulated	Regulates hypothalamic lipid sensing and energy homeostasis; decreased expression suggests impaired fatty-acid oxidation and metabolic signaling [75]
***Apoe***	−1.7143	0.000031	↓ Downregulated	Contributes to hypothalamic lipid transport and anorexigenic signaling; decreased expression suggests disrupted energy-balance regulation [76]
***Fgf2***	−1.1324	0.000036	↑ Upregulated	Promotes neurogenesis and recovery after TBI [77].
***Igf1***	+0.4599	0.000141	↑ Upregulated	Promotes neuroendocrine signaling and neuronal survival; increased expression may indicate an adaptive trophic response. [78]
***Calm2***	−1.1803	0.000245	↓ Downregulated	Encodes a core Ca^2+^ sensor supporting calcium/calmodulin signaling in hypothalamic metabolic and secretory pathways; decreased expression suggests blunted calcium-dependent signaling [79]
***Oxtr***	−0.6948	0.00052	↓ Downregulated	Mediates hypothalamic oxytocin signaling involved in thermoregulation and energy balance; decreased expression suggests impaired oxytocin responsiveness [80]
***Gata3***	+0.004662	0.001644	↑ Upregulated	Acts as a transcriptional regulator of neuroendocrine differentiation programs; increased expression may reflect adaptive transcriptional remodeling [81].
***Ngf***	+0.3022	0.001874	↑ Upregulated	Supports hypothalamic neurotrophic signaling and stress-responsive plasticity; increased expression may indicate enhanced reparative or adaptive neurotrophic drive [82]
***Olig2***	−0.2729	0.002654	↓ Downregulated	Regulates oligodendrocyte lineage specification and hypothalamic gliogenesis; decreased expression indicates impaired glial differentiation and reduced reparative plasticity [83,84]
***Sox2***	−0.3057	0.002793	↓ Downregulated	Maintains hypothalamic neural progenitor identity and hypothalamic neuroendocrine regulatory programs; decreased expression indicates reduced neurogenic support and diminished cellular plasticity [85,86]
***Hif1α***	–0.3489	0.004877	↓ Downregulated	Regulates hypoxia adaptation and angiogenic response; decreased expression indicates suppressed oxygen-sensing pathways. [87,88,89]
***Cort***	+0.40538	0.013198	↑ Upregulated	Modulates hypothalamic corticotropin-releasing hormone output; increased expression may reflect compensatory restraint of stress-axis activation [90]
***Tert***	+0.6802	0.01679	↑ Upregulated	Supports cellular stress resilience and HPA-axis homeostasis; increased expression may represent a protective response to stress-related dysregulation [91]
***Avp***	−2.1365	0.026808	↓ Downregulated	Regulates hypothalamic feeding control and stress-axis signaling; decreased expression suggests weakened neuroendocrine output [92]

Note: Negative values indicate gene downregulation, while positive values indicate upregulation compared with sham control. Adjusted *p*-values were calculated to control for false discovery rate (FDR), with adj. *p* < 0.05 considered statistically significant.

**Table 2:** Log10 transformation value of gene expression in the hypothalamus between the chronic TBI and sham groups. The sham control group corresponds to a baseline value of 0 after transformation.

Gene	Log10 Transformed Value	Adjusted *p*-Value (Adj. *p*. Val)	Regulation	Functional Relevance (Selected References)
***Rad18***	−0.46702	0.000015	↓ Downregulated	Impaired DNA repair activity associated with chronic neuronal stress [93,94]
***Nfrkb***	−1.19131	0.000016	↓ Downregulated	Transcriptional regulator involved in immune signaling and chromatin remodeling [95]
***Apoe***	−1.57262	0.000063	↓ Downregulated	Decreased lipid transport and neuronal repair capacity [65].
***Fgf2***	−1.09038	0.000073	↓↓↓ Strongly downregulated	Loss of trophic signaling and impaired neuroregeneration [77]
***Ppara***	−1.00623	0.00032	↓ Downregulated	Suppressed metabolic and anti-inflammatory control [38,40].
***Calm2***	−1.3199	0.000325	↓ Downregulated	Reduced calcium/calmodulin signaling may disrupt synaptic regulation [96,97].
***Gata3***	0.49245	0.00074	↑↑↑ Strongly upregulated	Transcription factor promoting neuroendocrine differentiation [98].
***Ngf***	0.43413	0.000832	↑ Upregulated	Enhanced neurotrophic response to injury [82]
***Oxtr***	−0.61792	0.00144	↓ Downregulated	Impaired oxytocin receptor signaling affecting HPA axis balance [99].
***Hif1α***	0.51216	0.00713	↑ Upregulated	Reactivation of hypoxia-responsive signaling during chronic stress [100,101].
***Igf1***	0.63476	0.0155	↑ Upregulated	Neuroprotective and restorative growth factor activity [102].
***Avp***	−2.18589	0.0328	↓↓↓ Strongly downregulated	Reduced vasopressin expression indicates long-term HPA axis dysregulation [103].
***Crh***	0.28978	0.03687	↑ Upregulated	Regulates hypothalamic–pituitary–adrenal axis activation and stress-responsive neuroendocrine output; increased expression indicates enhanced stress-axis drive [104,105].

Note: Positive values indicate upregulation, and negative values indicate downregulation relative to the sham group. Adj. *p* < 0.05 was considered significant.

**Table 3:** Log10 transformation value of gene expression in the pituitary between the chronic and acute TBI groups. The sham control group corresponds to a baseline value of 0 after transformation.

Gene	Log10 Transformed Value	Adjusted *p*-Value (Adj. *p*. Val)	Regulation	Functional Relevance (Selected References)
***Ptgs1 (cox1)***	0.21023	0.001758	↑ Upregulated	Encodes cyclooxygenase-1, involved in prostaglandin synthesis and inflammatory response [106].
***Rad18***	0.23979	0.008163	↑ Upregulated	DNA damage tolerance and post-replicative repair factor; elevated expression reflects increased genomic maintenance activity [93,94].
***Nfrkb***	0.25937	0.01258	↑ Upregulated	Transcriptional regulator implicated in immune and stress-response pathways [95].
***Tert***	0.2682	0.019901	↑ Upregulated	Supports telomere maintenance and cell survival programs; increased expression may indicate an adaptive proliferative or repair response in pituitary tissue [107,108].
***Ppara***	0.16372	0.020103	↑ Upregulated	Regulates lipid-sensing transcription and metabolic control of pituitary hormone expression; increased expression indicates enhanced fatty acid–responsive metabolic adaptation [109,110]
***Oxtr***	+0.4048	0.020225	↑ Upregulated	Mediates oxytocin responsiveness in pituitary lactotrophs; increased expression indicates enhanced prolactin-linked secretory sensitivity [111].
***Ngf***	0.19317	0.035901	↑ Upregulated	Supports pituitary neurotrophic and paracrine signaling and is secreted by anterior pituitary cells; increased expression indicates enhanced secretory and stress-responsive activity [112,113].

**Table 4:** Log10 transformation value of gene expression in the adrenal following TBI: comparison between acute phase and sham controls. The sham control group corresponds to a baseline value of 0 after transformation.

Gene	Log10 Transformed Value	Adjusted *p*-Value (Adj. *p*. Val)	Regulation	Functional Relevance (Selected References)
***Hif1α***	0.35518	0.000014	↑ Upregulated	Key regulator of cellular adaptation to hypoxia; activation indicates increased oxygen-sensing activity [101].
***Apoe***	0.317	0.00007	↑ Upregulated	Lipid transport and neuronal repair protein; supports membrane remodeling and oxidative stress defense [96]
***Ptgs2 (cox-2)***	−0.36596	0.000232	↓ Downregulated	Cyclooxygenase-2 involved in prostaglandin synthesis; suppression reflects transient anti-inflammatory response [106].
***Oxt***	−0.4647	0.000274	↓ Downregulated	Encodes oxytocin; decreased levels suggest reduced neuropeptide-mediated stress signaling [99].
***Ppara***	0.3263	0.000415	↑ Upregulated	Nuclear receptor regulating lipid metabolism and inflammation; its activation promotes metabolic recovery [38,40].
***Rad18***	0.20449	0.002745	↑ Upregulated	DNA repair and replication factor; upregulation indicates increased genomic maintenance under stress [93,94].
***Sox2***	−0.20912	0.015503	↓ Downregulated	
***Ptgs1 (cox-1)***	−0.1996	0.01924	↓ Downregulated	Constitutive cyclooxygenase enzyme; decreased expression reflects reduced basal prostaglandin activity [106].
***Calm2***	0.18318	0.01975	↑ Upregulated	Encodes calmodulin, a core Ca^2+^ sensor in adrenal cells; increased expression indicates enhanced calcium-dependent steroidogenic signaling [114].

**Table 5:** Log10 transformation value of gene expression in the adrenal gland following traumatic brain injury: comparison between chronic phase and sham controls. The sham control group corresponds to a baseline value of 0 after transformation.

Gene	Log10 Transformed Value	Adjusted *p*-Value (Adj. *p*. Val)	Regulation	Functional Relevance (Selected References)
***Agtr2***	+0.27529	0.042946	↑ Upregulated	Angiotensin II receptor type 2; mediates neuroprotective and vasodilatory responses [69].
***Gata3***	−0.1967	0.001449	↓ Downregulated	Regulates adrenal chromaffin cell differentiation and survival; decreased expression indicates impaired sympathoadrenal maintenance [115].
***Pomc***	−0.2428	0.003199	↓ Downregulated	Provides melanocortin-derived trophic support for adrenal corticosterone production; decreased expression indicates reduced steroidogenic drive [116].
***Apoe***	0.17413	0.010422	↑ Upregulated	Regulates adrenal cholesterol storage and trafficking; increased expression indicates enhanced cholesterol sequestration and restrained steroidogenic flux [117].
***Hif1α***	0.08635	0.011217	↑ Upregulated	Coordinates hypoxia-responsive control of adrenal steroidogenesis; increased expression indicates activated oxygen-sensing and steroidogenic adaptation pathways [118].
***Ppara***	0.24544	0.017408	↑ Upregulated	Regulates lipid metabolism and mitochondrial function; its activation supports cellular energy balance [38,40].
***Avp***	−0.10162	0.031612	↓ Downregulated	Regulates adrenal function through local autocrine/paracrine signaling and steroid secretory control; decreased expression indicates reduced vasopressin-mediated adrenal responsiveness [103,119].

**Table 6:** Log10 transformation value of gene expression in the adrenal gland following traumatic brain injury: comparison between acute phase and chronic phase. The sham control group corresponds to a baseline value of 0 after transformation.

Gene	Log10 Transformed Value	Adjusted *p*-Value (Adj. *p*. Val)	Regulation	Functional Relevance (Selected References)
***Hif1α***	−0.26883	0.00018	↓ Downregulated	Hypoxia-inducible factor-1α; reduced expression indicates decreased hypoxic signaling during the chronic recovery phase [101].
***Fgf2***	0.21108	0.001647	↑ Upregulated	Fibroblast growth factor 2; supports tissue remodeling and regeneration after sustained stress [77].
***Rad18***	−0.17229	0.004365	↓ Downregulated	E3 ubiquitin ligase involved in DNA repair; decreased expression reflects attenuation of DNA damage responses [93,94].
***Apoe***	−0.14287	0.018273	↑ Upregulated	Supports adrenal cholesterol homeostasis and esterified cholesterol accumulation; decreased expression indicates reduced cholesterol storage control with greater diversion toward steroid production [120].

## Data Availability

The original contributions presented in this study are included in the article/Supplementary Materials. Further inquiries can be directed to the corresponding author.

## Supplementary Materials

The following supporting information can be downloaded at https://www.mdpi.com/article/10.3390/ijms27125172/s1.

## Abbreviations

**ACTB**	Beta-actin (Housekeeping gen: *Actb*)
**ACTH**	Adrenocorticotropic hormone
**AGTR2**	Angiotensin II receptor type 2 (Gene: *Agtr2*)
**APOE**	Apolipoprotein E (Gene: *Apoe*)
**AVP**	Arginine vasopressin (Gene: *Avp*)
**BDNF**	Brain-derived neurotrophic factor (Gene: *Bdnf*)
**CALM2**	Calmodulin 2 (Gen: *Calm2*)
**CCI**	Controlled cortical impact
**cDNA**	Complementary DNA
**CNS**	Central nervous system
**COX1**	Cyclooxygenase-1 (Gene: *Ptgs1*)
**COX2**	Cyclooxygenase-2 (Gene: *Ptgs2*)
**CRH**	Corticotropin-releasing hormone (Gene: *Crh*)
**FDR**	False discovery rate
**FGF2**	Fibroblast growth factor 2 (Gene: *Fgf2*)
**GATA3**	GATA binding protein 3 (Gene: *Gata3*)
**HIF1A**	Hypoxia-inducible factor 1-alpha (Gene: *Hif1a*)
**HPA**	Hypothalamic–pituitary–adrenal
**IGF1**	Insulin-like growth factor 1 (Gene: *Igf1*)
**logFC**	Log10 fold change
**mRNA**	Messenger ribonucleic acid
**NGF**	Nerve growth factor (Gene: *Ngf*)
**OLIG2**	Oligodendrocyte transcription factor 2 (Gene: *Olig2*)
**OXT**	Oxytocin (Gene: *Oxt*)
**OXTR**	Oxytocin receptor (Gene: *Oxtr*)
**POMC**	Proopiomelanocortin (Gene: *Pomc*)
**PPARA**	Peroxisome proliferator-activated receptor alpha (Gene: *Ppara*)
**qPCR**	Quantitative real-time PCR
**RAD18**	RAD18 E3 ubiquitin protein ligase (Gene: *Rad18*)
**SD**	Standard deviation
**SOX2**	SRY-box transcription factor 2 (Gene: *Sox2*)
**TBI**	Traumatic brain injury
**TERT**	Telomerase reverse transcriptase (Gene: *Tert*)
