# Supplementary material for: Neuronal Injury and Regeneration-Linked Gene Expression Dynamics in the Hypothalamic–Pituitary–Adrenal Axis Following Experimental Traumatic Brain Injury
Source: Int J Mol Sci. 2026 Jun 7;27(12):5172. doi: 10.3390/ijms27125172 (PMC13300581; doi:10.3390/ijms27125172)
Supplement: Supplementary file 1 [file ijms-27-05172-s001.zip › Supp figure revised 25.05.26.pdf]

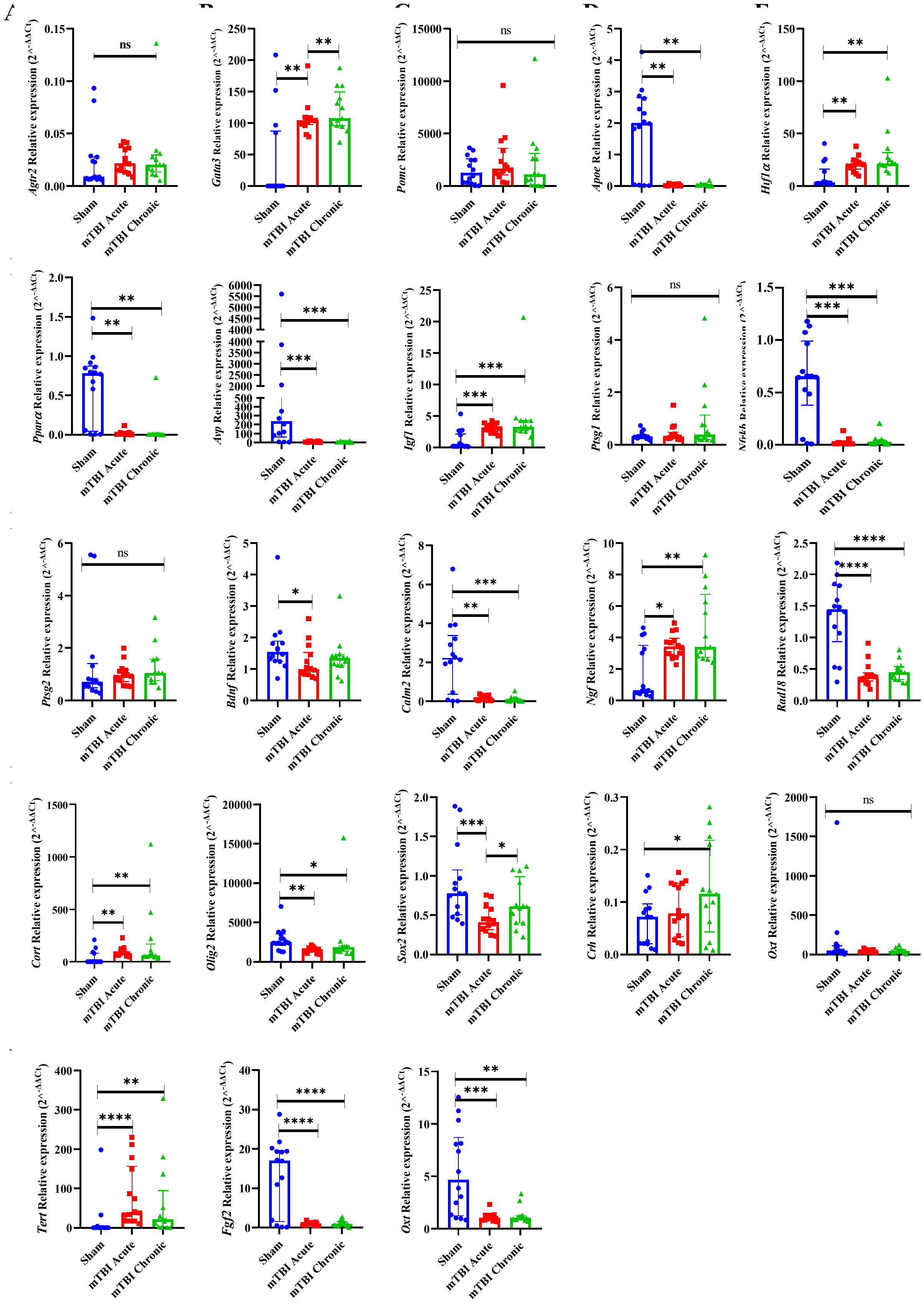

Supp Figure 1. Relative expression levels of *Agtr2, Gata3, Pomc, Apoe, Hif1a, Ppara, Avp, Igf1, Ptgs1, Bdnf, Nfrkb, Ptgs2, Calm2, Ngf, Rad18, Cort, Olig2, Sox2, Crh, Oxt, Tert, Fgf2,* and *Oxtr* genes in hypothalamus tissue (panels A–X, respectively). Gene expression levels were calculated using the 2^-ΔΔCt method and are presented as relative gene expression levels normalized to the housekeeping gene and control group. Data are presented in accordance with distribution properties: variables exhibiting normal distribution are expressed as mean ± standard deviation (SD), whereas non-normally distributed variables are presented as median

with interquartile range (IQR). Individual data points are shown for each sample. Statistical analyses were performed as appropriate for data distribution, and differences were considered statistically significant at $p < 0.05$.

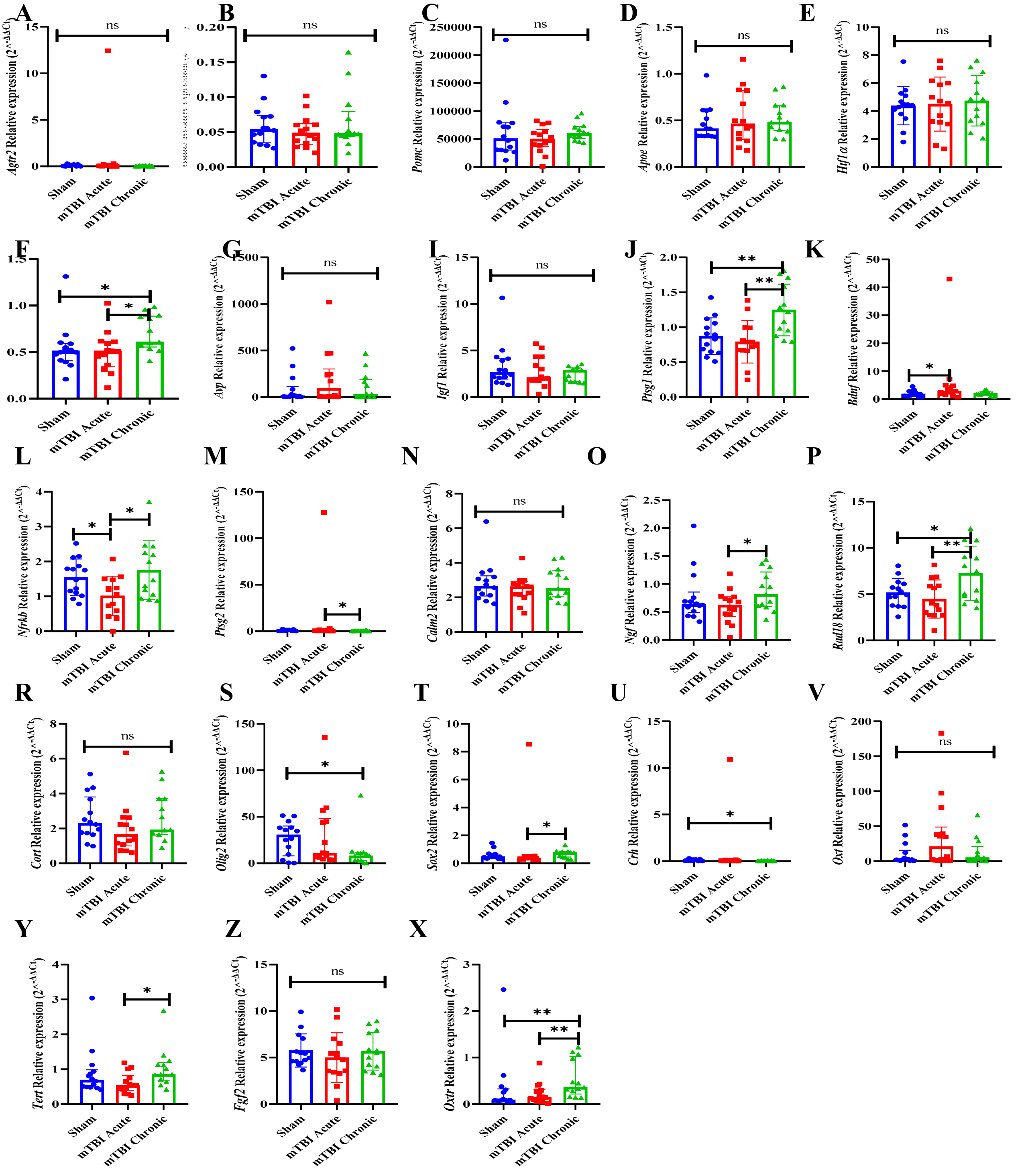

Supp Figure 2. Relative expression levels of *Agtr2, Gata3, Pomc, Apoe, Hif1a, Ppara, Avp, Igf1, Ptgs1, Bdnf, Nfrkb, Ptgs2, Calm2, Ngf, Rad18, Cort, Olig2, Sox2, Crh, Oxt, Tert, Fgf2,* and *Oxtr* genes in pituitary tissue (panels A–X, respectively). Gene expression levels were calculated using the 2^-ΔΔCt method and are presented as relative gene expression levels normalized to the housekeeping gene and control group. Data are presented in accordance with distribution properties: variables exhibiting normal distribution are expressed as mean ± standard deviation (SD), whereas non-normally distributed variables are presented as median with interquartile range (IQR).

Individual data points are shown for each sample. Statistical analyses were performed as appropriate for data distribution, and differences were considered statistically significant at $p < 0.05$.

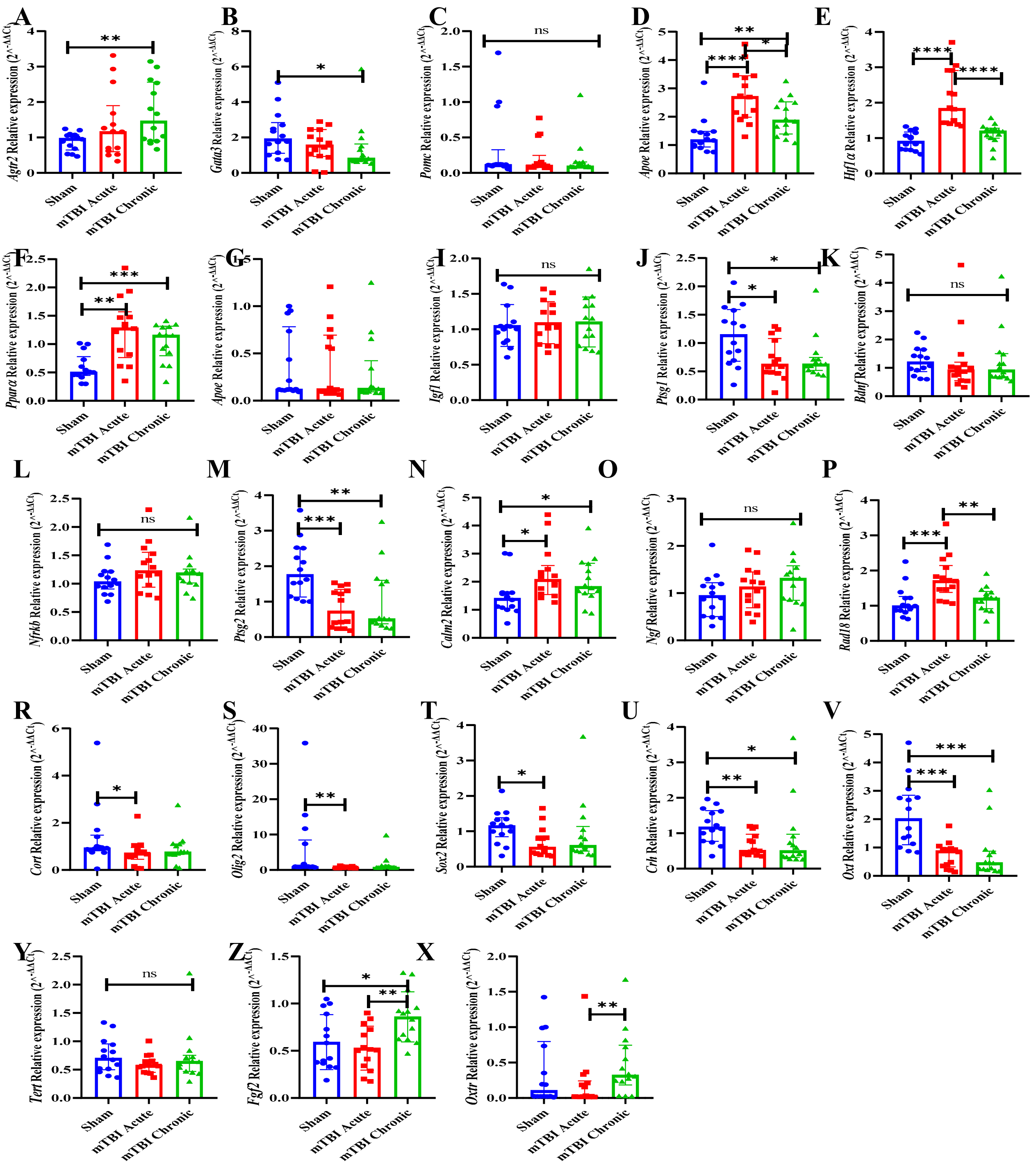

Supp Figure 3. Relative expression levels of *Agtr2, Gata3, Pomc, Apoe, Hif1a, Ppara, Avp, Igf1, Ptgs1, Bdnf, Nfrkb, Ptgs2, Calm2, Ngf, Rad18, Cort, Olig2, Sox2, Crh, Oxt, Tert, Fgf2,* and *Oxtr* genes in adrenal tissue (panels A–X, respectively). Gene expression levels were calculated using the 2^-ΔΔCt method and are presented as relative gene expression levels normalized to the housekeeping gene and control group. Data are presented in accordance with distribution properties: variables exhibiting normal distribution are expressed as mean ± standard deviation (SD), whereas non-normally distributed variables are presented as median with interquartile range (IQR).

Individual data points are shown for each sample. Statistical analyses were performed as appropriate for data distribution, and differences were considered statistically significant at $p < 0.05$.
